# A Plea for Speed in Operating

**Published:** 1899-08-26

**Authors:** 


### A PLEA FOR SPEED IN OPERATING.

Writing in the New York *Medical Record* on the subject of shock in modern surgery, Dr. Shrady puts in a plea for greater speed in the performance of operations. Much as we should like to believe to the contrary, he says, a great deal of the mortality rate after operative work is due to surgical shock which might possibly in many cases be prevented. Strangely as it may sound in this day of brilliant surgical triumphs, when nothing, however venturesome, in surgical technique appears beyond the sphere of realised demonstration, men seem nevertheless inclined to forget some of those fundamental principles in operative work which meant so much to those who have preceded us. Then it was that everything bent to speed in wielding the knife, and the shortest time employed in so doing meant the highest evidence of skill. Now it would appear that, with the present immunity from pain and wound infection, we are swinging to the extreme of wasting time and prolonging shock. The operator is tempted to trust too much to the staying power of his patient, and if this fails "the account is then squared by the simple statement that the patient was unable to survive the shock." By many it is believed that any additional shock is impossible so long as the mere pain of an operation is abolished. We must not forget, however, that shock is a composite condition, a jar to the equilibrium of the entire sympathetic system, in the production of which mere pain oftentimes plays an insignificant part. With ether and chloroform we save the patient from positive agony, but we are constantly wasting his nervous force of resistance even while securing him against obvious injury, for the prolonged administration of an anæsthetic is itself a cause of shock. We should then do all that is possible to shorten an operation. Of two methods of doing the same thing the one should be chosen which occupies the least time; if a grave operation can be done in two stages so much the better; and no encouragement should be given to the practice of doing several operations on a patient at one sitting. Then when the operation is done time must not be wasted over the "deliberate, tedious, and often fussy dressings" which some surgeons carry out "before the patient is warmly tucked in his bed." Very often, says Dr. Shrady, the matter of sewing up a large wound to escape union by second intention directly invites death by first intention. "We must acknowledge from a clinical point of view that, all other things being equal, those patients who are the shortest time on the operating-table have incomparably better chances for recovery than those who are subjected to the present fashionable details of tediously deliberate technique. . . Sometimes if the surgeon, while at work, thought more of his patient than of the operation the result might be very different." We think that Dr. Shrady's remarks contain a truth which should be constantly borne in mind. In fact, the necessity of shortening operations and saving shock is, we think, borne in mind and very earnestly expressed by some of our best surgeons. We do not think, however, that technique should be skimped, we do not think that wounds should be left, as Dr. Shrady suggests, only partially sewn up, or that dressings should be imperfectly applied; but we do think that in some cases surgeons might, by practising beforehand every detail of what has to be done, by sharing the labour of "sewing up," and by the use of various mechanical devices in place of the beloved roller bandage, save much time in many operations. It has always seemed to us that much of the success which seemed to attend the introduction of the Murphy button was due to the saving of time which it made possible, and we have no doubt at all that the success which falls to the lot of some silent surgeons, who go straight at their work and do it deftly, without a moment's interruption for either demonstration or consultation, is largely due to the lessened length of operation which results.

